# Reproducibility of cuff-induced hyperemic popliteal artery flow analysis using phase-contrast MRI: Patients With Intermittent Claudication Injected With ALDH Bright Cells (PACE) trial

**DOI:** 10.1186/1532-429X-18-S1-P359

**Published:** 2016-01-27

**Authors:** Tomoki Fujii, Chikara Noda, Victor Nauffal, Elzbieta H Chamera, Joao A Lima, Emerson C Perin, Alan T Hirsch, Bharath Ambale Venkatesh

**Affiliations:** 1 Johns Hopkins University, Baltimore, MD USA; 2 Showa University Hospital, Tokyo, Japan; 3 Texas Heart Institute, Houston, TX USA; 4 Clinical and Translational Science Institute at University of Minnesota, Minneapolis, MN USA

## Background

Peripheral artery disease (PAD) is a major consequence of atherosclerosis. Recently, quantitative evaluation of hyperemic arterial flow reserve measured using cuff occlusion has been identified as a method of measuring arterial stiffness. Phase-contrast MRI (PCMRI) is an extensively used and accurate technique to quantify vessel flow. The aim of this study was to assess the inter-observer, intra-observer and test-retest reproducibility of hyperemic popliteal arterial flow measurements using PCMRI in a protocol developed for the Cardiovascular Cell Therapy Research Network (CCTRN).

## Methods

16 healthy volunteers underwent PCMRI of the popliteal artery using cuff compression at the thigh in order to provoke calf hyperemia. 8 participants were scanned on a 1.5T Siemens Avanto scanner and 8 participants on a 3T Siemens Trio scanner. All participants had a repeat MRI examination in the same scanner with the same protocol for the test-retest reproducibility. The PCMRI was performed using the following parameters: TR/TE/flip angle = 20.00 msec/2.18 msec/25°, field of view (FOV) = 38 cm to cover both legs; slice thickness = 5 mm; matrix = 256 × 256; NEX = 1; Phase encoding direction = A>>P; parallel imaging factor = 2; and time of acquisition per scan = 30-40 s; VENC = 100 cm/s. PCMRI of the popliteal artery at resting state and hyperemic state was performed repeatedly in conjunction with cardiac gating. Initially, participants had 3 PCMRI scans before the cuff was inflated to measure resting flow. Subsequently, the cuff was inflated to the occlusion pressure (calculated as systolic arm pressure + 50 mmHg) and kept inflated for 5 minutes. After 5 minutes, we simultaneously deflated the cuff and measured flow through PCMRI's for 10 minutes with the same imaging parameters. Qflow (Medis, Netherlands) was used for analysis of peak arterial flow of each scan. The peak resting flow was measured as the average of the 3 scans pre-inflation. The peak hyperemic flow was the peak flow post-deflation at the time-to-peak hyperemic flow. Arterial flow reserve was calculated as the difference of hyperemic and resting flows. For the statistical analysis, interclass correlation coefficient (ICC) was used to measure the agreement of test-retest, inter- and intra-study reproducibility.

## Results

The mean of age of the participants were 52 ± 5 years; range, 44 - 59 years (11 men and 5 woman). The flow measurements of the peak resting flow, peak hyperemic flow, arterial flow reserve and time to peak at the popliteal artery using PC MRI had excellent inter- and intra-observer reproducibility as seen by the ICC (range 0.83- 1.00).

## Conclusions

The test-retest reproducibility was good to excellent for peak resting and hyperemic flow values but moderate to good for arterial flow reserve and time-to-peak flow. Hyperemic arterial flow measurement may be a useful means to measure arterial stiffness.

**Table 1:** Reproducibility of popliteal arterial flow analysis using Phase-contrast MRI in the 16 participants

Inter Study	Parameters	Reader 1	Reader 2	Mean diference	ICC
	Peak Resting Flow (ml/s)	6.57 ± 2.78	6.62 ± 3.42	0.04 ± 1.04	0.96**
	Peak Hyperemic Flow (ml/s)	13.5 ± 6.10	13.3 ± 6.23	-0.14 ± 2.31	0.94**
	Arterial Flow Reserve (ml/s)	6.92 ± 4.21	6.74 ± 3.38	-0.18 ± 2.34	0.83**
	Time To Peak (sec)	34.9 ± 67.6	41.4 ± 57.0	6.50 ± 18.8	0.98**
**Intra Study**	Parameters	Time 1	Time 2	Mean diference	ICC
	Peak Resting Flow (ml/s)	6.23 ± 2.87	6.26 ± 3.00	0.03 ± 0.95	0.95**
	Peak Hyperemic Flow (ml/s)	13.5 ± 7.02	13.31 ± 6.19	-0.22 ± 0.85	0.98**
	Arterial Flow Reserve (ml/s)	7.30 ± 4.90	7.05 ± 4.15	-0.26 ± 1.23	0.93**
	Time To Peak (sec)	17.3 ± 6.86	17.3 ± 6.86	0.00 ± 0.00	1.00**
**Test-Retest Study**	Parameters	Visit 1	Visit 2	Mean diference	ICC
	Peak Resting Flow (ml/s)	6.23 ± 2.87	6.57 ± 2.78	0.35 ± 0.94	0.94**
	Peak Hyperemic Flow (ml/s)	13.5 ± 7.02	13.5 ± 6.10	-0.03 ± 3.55	0.85**
	Arterial Flow Reserve (ml/s)	7.30 ± 4.90	6.92 ± 4.21	-0.39 ± 3.00	0.74*
	Time To Peak (sec)	17.3 ± 6.86	34.9 ± 67.6	17.6 ± 67.8	0.79*

Value are Mean ± SD, SD: standard deviation, ICC: intraclass correlation coefficient. ** = p < 0.001, * = p < 0.01.

**Figure 1:**
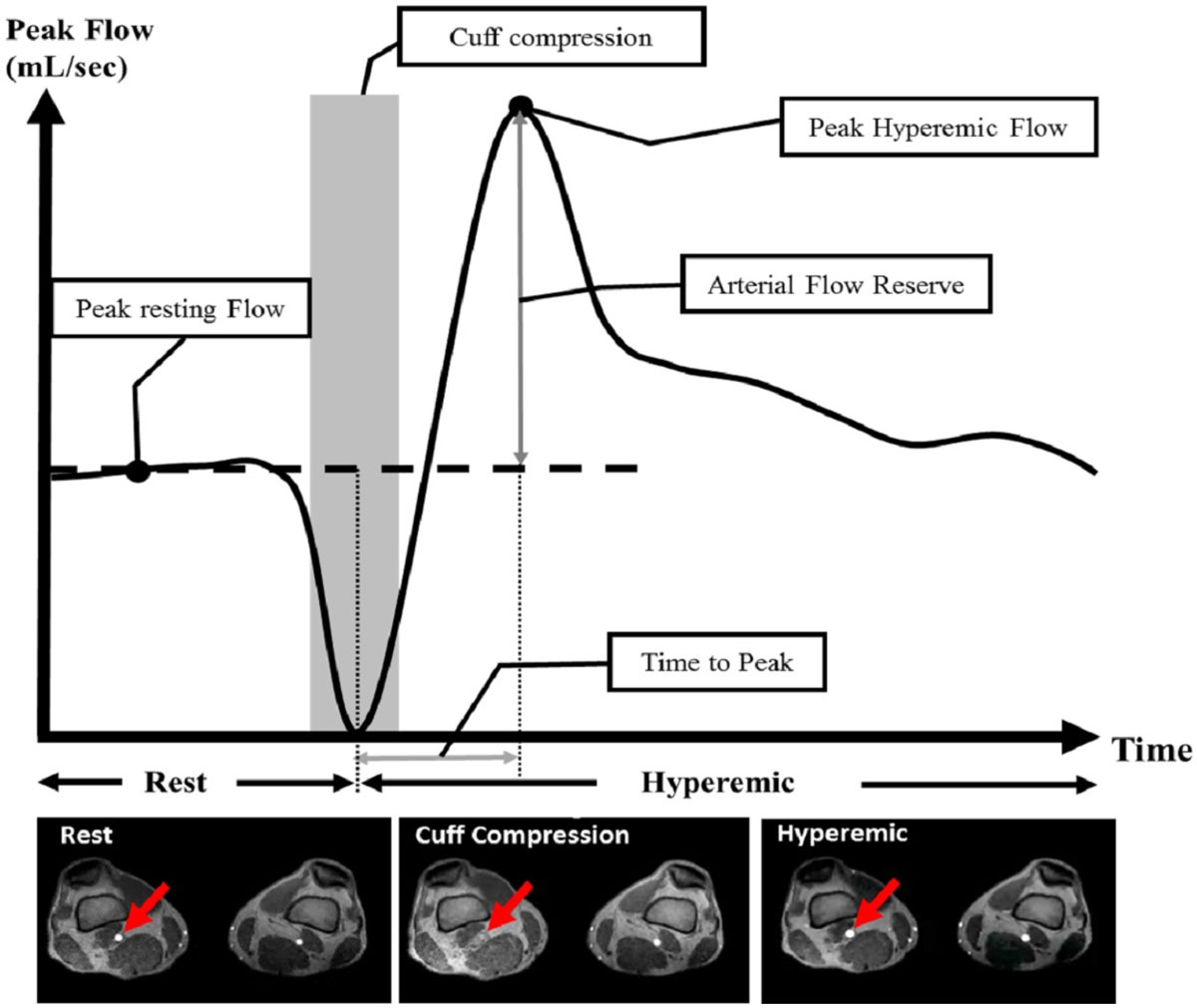
**Overview of the Flow measurement - Red arrows indicate the popliteal artery which is measured peak flow**. Popliteal arterial flow at the Cuff compression is total occlusion.

